# Efficient Bioactive Surface Coatings with Calcium Minerals: Step-Wise Biomimetic Transformation of Vaterite to Carbonated Apatite

**DOI:** 10.3390/biomimetics9070402

**Published:** 2024-07-02

**Authors:** Dong Hyun Kim, Ki Ha Min, Seung Pil Pack

**Affiliations:** 1 Department of Biotechnology and Bioinformatics, Korea University, Sejong 30019, Republic of Korea; jklehdgus@korea.ac.kr; 2 Institute of Industrial Technology, Korea University, Sejong 30019, Republic of Korea; alsrlgk@gmail.com

**Keywords:** biocoating, biomimetic transformation, calcium phosphate, calcium carbonate, osteogenesis

## Abstract

Carbonated apatite (CAp), known as the main mineral that makes up human bone, can be utilized in conjunction with scaffolds to increase their bioactivity. Various methods (e.g., co-precipitation, hydrothermal, and biomimetic coatings) have been used to provide bioactivity by forming CAp on surfaces similar to bone minerals. Among them, the use of simulated body fluids (SBF) is the most popular biomimetic method for generating CAp, as it can provide a mimetic environment. However, coating methods using SBF require at least a week for CAp formation. The long time it takes to coat biomimetic scaffolds is a point of improvement in a field that requires rapid regeneration. Here, we report a step-wise biomimetic coating method to form CAp using calcium carbonate vaterite (CCV) as a precursor. We can manufacture CCV-transformed CAp (V-CAp) on the surface in 4 h at least by immersing CCV in a phosphate solution. The V-CAp deposited surface was analyzed using scanning electron microscopy (SEM) images according to the type of phosphate solutions to optimize the reaction conditions. X-ray diffraction (XRD) and attenuated total reflection-Fourier transform infrared (ATR-FTIR) analysis validated the conversion of CCV to V-CAp on surfaces. In addition, the bioactivity of V-CAp coating was analyzed by the proliferation and differentiation of osteoblasts in vitro. V-CAp showed 2.3-folded higher cell proliferation and 1.4-fold higher ALP activity than the glass surface. The step-wise method of CCV-transformed CAp is a biocompatible method that allows the environment of bone regeneration and has the potential to confer bioactivity to biomaterial surfaces, such as imparting bioactivity to non-bioactive metal or scaffold surfaces within one day. It can rapidly form carbonated apatite, which can greatly improve time efficiency in research and industrial applications.

## 1. Introduction

Bones are vital organs that support the body, protect internal organs, and store ions [1]. In order to repair bone damage caused by an accident, it is necessary to use a non-infectious material that can be used without restriction [2]. The prevailing approach for the treatment of damaged bones is to use materials with low bioactivity, such as titanium-based pins and screws, to stimulate bone regeneration [3]. Scaffolds and supporting structures for bone regeneration require three conditions: (1) bone-like biological and mechanical properties, (2) the ability to fabricate individual geometries and multi-level pore structures of bone scaffolds, and (3) the necessity of enabling the transition from artificial to biological structures [4]. Several studies are underway to enhance the bioactivity of bone cells and scaffolds [5,6,7].

One approach is to coat the surface of the scaffold with biominerals to provide a bioactive environment that helps induce rapid bone regeneration [8]. The use of carbonated apatite, such as hydroxyapatite (Ca_10_(PO_4_)_6_(OH)_2_), a major mineral phase found in human hard tissue, to provide a human-like environment is another option [9]. Carbonated apatite can promote the proliferation and differentiation of bone cells. It can also interact with collagen to induce mineralization [10]. Mondal et al. fabricated a scaffold coated with hydroxyapatite, which is derived from fish. This biomineral coating on the scaffold showed a better and faster healing rate for new bone formation in vivo [11]. The synthesis, characterization, and analysis of carbonated apatite for its use as an implantable osteocompatible biomaterial has also been studied [12,13,14].

Methods for synthesizing carbonated apatite (CAp), such as hydroxyapatite, include co-precipitation [15], sol–gel [16], microwave-assisted synthesis [17], glass transition [18], template-directed [19], and hydrothermal reactions [20]. One method for incorporating carbon dioxide into precipitated solids involves replacing phosphate ions with carbonate ions. This results in CAp with a low carbonate content and a non-stoichiometric Ca/P ratio [21]. Nevertheless, CAp, with a stoichiometrically low Ca/P ratio, is inert at high temperatures, which is typical of ceramic sintering or plasma spraying. This property prevents decomposition into lime or tricalcium phosphate (TCP) [22]. CAp, which contains many carbonates, can be synthesized via multi-step methods, such as precipitating soluble calcium salts (Ca^2+^) as PO_4_^3−^ in the presence of CO_3_^2−^ (liquid route) [23] or heating calcium phosphate above 1000 °C in the presence of a carbon dioxide stream (thermal route) [24]. These methods have the disadvantage of requiring additional manipulations to remove waste counterions or a heating step under controlled carbon dioxide [21].

A widely used biomimetic method to promote biocompatibility and osteointegration of implantable materials is biomimetic coating [25]. A concept introduced by Kokubo et al. in 1990, in which metal implants were coated with an apatite layer through immersion in simulated body fluid (SBF) [26]. In general, it reduces unfavorable immune responses and promotes a more favorable environment for bone regeneration. This is accomplished through improved biocompatibility, release of favorable ions, enhanced recruitment and differentiation of osteogenic cells, and modulation of the immune response toward a healing phenotype [27,28]. However, the application of this biomimetic technique has been limited by the long immersion time (more than 1 week) and the need for active chemical groups to form the apatite layer [29]. To overcome the limitations, Feng et al. used a precalcification method, which resulted in a reduction of the formation time to three days [30]. Moreover, a shorter CAp preparation time is expected to be beneficial for in situ use of cells with biomimetic CAp during the coating process [31]. Accordingly, this study aimed to develop a method for the fabrication of calcium carbonate as a precursor to accelerate the generation of CAp in a shorter amount of time.

Calcium carbonate is a mineral that can exist in various forms (e.g., aragonite, calcite, and vaterite). Among them, calcium carbonate vaterite (CCV) has a porous spherical shape and exhibits high specific surface area, solubility, and porosity compared to calcite and aragonite [32,33,34]. Recently, CCV has been investigated as a potential material for bone regeneration due to its biocompatibility and physicochemical properties [33,35]. In particular, CCV can provide a mimetic environment that can provide the necessary components for natural bone production in vivo [36,37]. These properties of CCV may be advantageous for bone regeneration when used as biomaterials [38].

**Figure 1:**
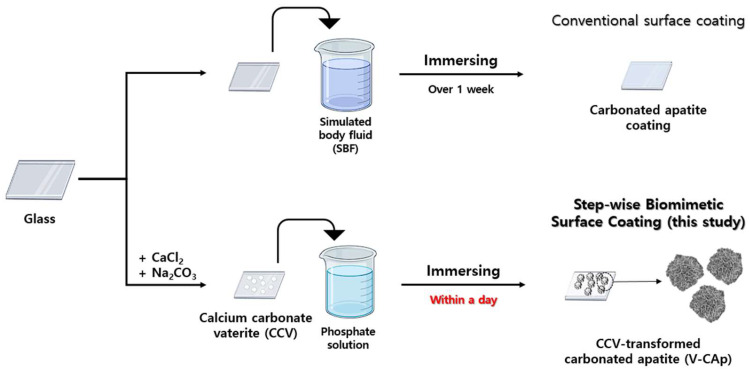
Schematic illustration of step-wise biomimetic surface coating (this study) compared to surface coating with SBF (conventional). The CCV was synthesized using the spontaneous precipitation method. The CCV was immersed in a phosphate solution to synthesize V-CAp, which is an immersing method. The objective of this study was to investigate the transformation of V-CAP in response to variations in immersion time and phosphate solution type.

In this study, we developed a step-wise method using calcium carbonate vaterite (CCV) as a precursor for CAp generation (Figure 1). This is a kind of biomimetic process to form CAp by combining CCV (as calcium ion sources) with a phosphate solution (as phosphate ion sources). The transformation of CCV to CAp was carried out in a shorter time compared to the method of generating CAp with SBF. The effect of CCV-transformed CAp (V-CAp) on osteoblast cell proliferation and bone differentiation formation in vitro was investigated. The V-CAp coating produced using the step-wise biomimetic method shows promising potential for use in bone regeneration-inducing materials and scaffolds.

## 2. Materials and Methods

### 2.1. Materials

Materials and reagents, including calcium chloride dehydrate (CaCl_2_), were purchased from Sigma Aldrich (St. Louis, MO, USA). Sodium carbonate anhydrous (Na_2_CO_3_) was procured from Duksan Pure Chemicals (Ansan, Republic of Korea), and microscope slides (25 mm in width, 75 mm in length, and 1 mm in thickness) were purchased from Marienfeld Superior (Lauda-Königshofen, Germany). Additionally, a 1 N sodium hydroxide (NaOH) standard solution was purchased from Daejung Reagent Chemicals (Siheung, Republic of Korea). All other chemical reagents utilized were of analytical grade.

### 2.2. Glass Cleaning

A glass slide was used as a template for coating with calcium carbonate and carbonated apatite. The purpose of cleaning the glass was to remove any organic contamination. The glass was cut into pieces measuring 10 mm × 10 mm × 1 mm and washed using a US cleaner (Branson Ultrasonics, Danbury, CT, USA) containing 20% ethanol and deionized water (D.W). After washing, the glass was immersed in a 1 N NaOH standard solution for 1 h and rinsed with D.W.

### 2.3. Preparation of Calcium Carbonate Vaterite (CCV)

Calcium carbonate vaterite (CCV) was synthesized using the spontaneous precipitation method [39]. A solution of 100 mM CaCl_2_ was added to a 250 mL beaker, and a cleaned glass was placed at the bottom of the beaker. Subsequently, 100 mM Na_2_CO_3_ was added without mixing during the reaction. The volume ratio of CaCl_2_ to Na_2_CO_3_ was 1:1. After a 15-min reaction at 25 °C, the glass surface was coated with CCV. The CCV-coated glass was removed from the solution and washed with ethanol and D.W, and then thoroughly dried. The CCV was dried in the oven (60 °C) for 1 h and then stored at room temperature. CCV was used as a carbonated apatite-transforming precursor.

### 2.4. Transformation of Carbonated Apatite (V-CAp) from Calcium Carbonate Vaterite (CCV)

Carbonated apatite from vaterite (V-CAp) was synthesized using the immersion method to transform the CCV deposited on the surface [40]. The effect on carbonated apatite formation depends on the components of the phosphate solution; thus, the phosphate solution was prepared as follows [41]. A 50 mM phosphate buffer containing 100 mM NaCl at pH 7.6 was prepared. Phosphate solution type 1 (PS-1) and phosphate solution type 2 (PS-2) were synthesized using 50 mM monosodium phosphate with 100 mM NaCl and 50 mM disodium phosphate with 100 mM NaCl, respectively. Both phosphate solutions were adjusted to pH 7.6. Commercial phosphate buffer saline (PBS, SAMCHUN, Seoul, Republic of Korea) and simulated body fluid (SBF, BIOSESANG, Incheon, Republic of Korea) were also prepared for comparing the transform V-CAp (Table 1) [31,42]. The glass coated with CCV was immersed in the phosphate buffer for 2 to 16 h at 37 °C to transform CCV into carbonated apatite (V-CAp) [31]. After the reaction, the glass coated with V-CAp was removed from the phosphate buffer, washed with D.W and ethanol, and dried. All experiments were performed with V-CAp formed after 16 h of reaction. The surface roughness and size of CCV and V-CAp were analyzed using Image J software (Version 1.54).

### 2.5. Characterization of Carbonated Apatite

The morphology of the calcium minerals (CCV, CAp, and V-CAp) was analyzed using scanning electron microscopy (SEM) (KBSI; Jeonju, Republic of Korea) coupled with energy dispersive X-ray spectroscopy (EDS) for elemental analysis. The structure of CCV and V-CAp was analyzed through X-ray diffraction (XRD) at the Korea Basic Science Institute (KBSI; Daegu, Republic of Korea) using a Panalytical Empyrean diffractometer with Cu-Kα radiation (λ_avg_ = 1.5425 Å), operating at 40 kV and 25 mA. The diffraction spectra of powder samples were collected from 10° to 80° of 2θ at a scan rate of 0.04°/s. Attenuated total reflection-Fourier transform infrared (ATR-FTIR) spectroscopy of CCV and V-CAp was performed using an FT-IR spectrophotometer (Perkin Elmer; Waltham, MA, USA).

### 2.6. In Vitro Cell Culture Experiments

Mouse osteoblast MC3T3-E1 subclone four cells (ATCC^®^ CRL-2593^TM^; Manassas, VA, USA) from passage 25 were cultured in a growth medium containing Minimal Essential Medium alpha (MEM-α; GE Healthcare HyClone™, Marlborough, MA, USA) with 10% FBS (fetal bovine serum) and 1% antibiotics at 37 °C with 5% CO_2_. Osteogenic differentiation was induced using a bone differentiation medium (growth medium containing 50 µg mL^−1^ ascorbic acid and 10 mM β-glycerophosphate). During cell culture and differentiation, the medium was changed every 3 days.

### 2.7. Cell Proliferation Assay

Adhesion and proliferation of osteoblast cells were confirmed using the prepared sample. MC3T3-E1 cells (1.0 × 10^4^ cells/well) were seeded onto glass, glass coated with CCV, and glass coated with V-CAp placed in a 24-well culture plate. The cells were incubated at 37 °C in a 5% CO_2_ incubator for 3 and 7 days. During the cell culture and differentiation process, the medium was changed every 3 days. Cell proliferation was measured using a 3-(4,5-dimethylthiazol-2-yl)-5-(3-carboxymethoxyphenyl)-2-(4-sulfophenyl)-2H-tetrazolium (MTS) assay with the CellTiter 96^®^ Aqueous One Solution Cell Proliferation assay kit (Promega, Fitchburg, WI, USA). The reaction solution from the MTS assay was transferred to a 96-well plate, and the absorbance was measured using a microplate reader (Infinite M200 PRO, TECAN, Zürich, Switzerland) at 490 nm.

### 2.8. Cell Differentiation Assay

MC3T3-E1 cells (1.0 × 10^4^ cells/well) were seeded onto glass, glass coated with CCV, and glass coated with V-CAp in a 24-well culture plate. The cells were incubated at 37 °C in a 5% CO_2_ incubator for 1 week. The medium was changed to a differentiation medium (same as above), and the ALP activity was measured after 7 and 14 days. The medium was refreshed every 3 days. Alkaline phosphatase (ALP) activity was measured using the p-nitrophenyl phosphate (pNPP) liquid substrate system (Sigma Aldrich, Taunton, MA, USA). The cell-cultured samples were washed with 500 µL of phosphate buffer saline (PBS). The glasses were transferred to a new 24-well plate and lysed with 200 µL of PBS containing 1% Triton X-100 for 5 min. After cell lysis, 200 µL of pNPP solution was added, followed by incubation at 37 °C for 30 min. The reaction solution from the ALP assay was transferred to a 96-well plate, and the absorbance was measured using a microplate reader (Infinite M200 PRO, TECAN, Zürich, Switzerland) at 405 nm.

### 2.9. Statistical Analysis

All values related to the experiments of the calcium minerals (CCV, CAp, and V-CAp) are presented as the mean ± standard deviation (SD). Statistical analyses were performed using the Student’s *t*-test for pairwise comparisons between two groups of interest, and two-way or three-way analysis of variance (ANOVA) with Fisher’s least significant difference (LSD) post hoc test for multiple comparisons. Differences with *p*-values below 0.05 were considered statistically significant.

## 3. Results and Discussion

### 3.1. Morphological Analysis of Vaterite-Transformed Carbonated Apatite (V-CAp)

**Figure 2:**
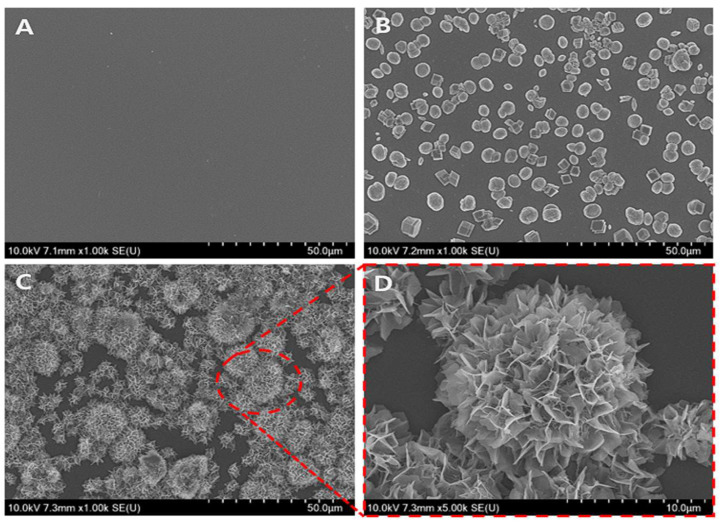
The morphology of CCV and V-CAp on template surface coating: (**A**) a glass surface, (**B**) CCV coating on a glass surface, and (**C**) V-CAp coating on a glass surface. (**D**) High magnification of V-CAp. CCV was produced using the spontaneous precipitation method, while V-CAp was produced through a 16-h immersion time at 37 °C.

The bioactive surface was coated using a step-wise method to form carbonated apatite (CAp) from calcium carbonate vaterite (CCV). The first step was to synthesize a highly bioactive CCV under supersaturated conditions. The SEM image in Figure 2 provides a representative overview of the coated surface with CCV-transformed CAp (V-CAp). The glass surface (Figure 2A) was coated with CCV after synthesis through spontaneous precipitation (Figure 2B). The CCV showed micro-sized spheres with a size distribution from 4 to 6 μm. The transformation to V-CAp was performed by immersing CCV in a phosphate solution (Figure 2C), resulting in a flower-like structure with V-CAp sizes ranging from 5 to 20 μm. A higher magnification SEM image of V-CAp is provided in Figure 2D. Phosphate ions interacted with calcium ions from CCV, leading to the generation of CCV-transformed CAp (V-CAp).

**Figure 3:**
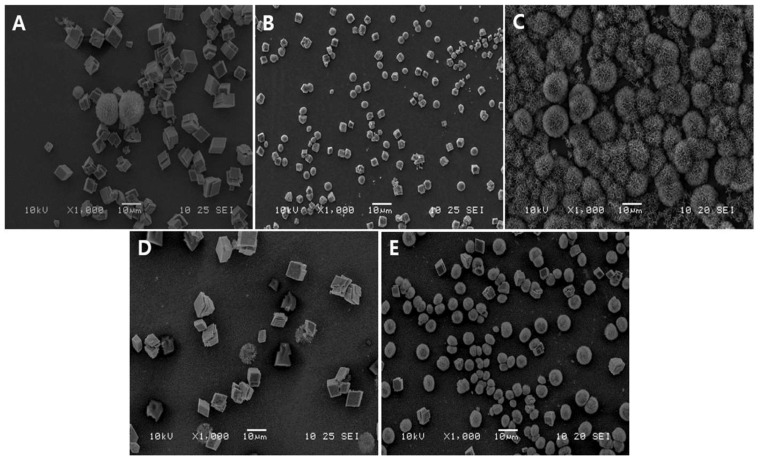
SEM image of V-CAp formation depending on phosphate solution: (**A**) phosphate buffer (P buffer), (**B**) phosphate solution type 1 (PS-1), (**C**) phosphate solution type 2 (PS-2), (**D**) phosphate buffer saline (PBS), and (**E**) simulated body fluid (SBF). The pH of P buffer, PS-1, and PS-2 was adjusted to 7.6, while the pH of PBS was 7.6 and that of SBF was 6.7. The CCV was immersed in the prepared phosphate solutions for 16 h at 37 °C.

Figure 3 shows the SEM images of V-CAp formation using different types of phosphate solutions after 16 h of immersion. Figure 3A–C show the transformation of CCV to CAp using phosphate buffer (P buffer), phosphate solution type 1 (PS-1), and phosphate solution type 2 (PS-2), respectively. As shown in Figure 3B, the morphology of CCVs after immersion in PS-1 was not significantly different from before. CCV with PS-2 showed a significant transformation to V-CAp (Figure 3C). However, CAp formation in P buffer (Figure 3A), commercial PBS (Figure 3D), and SBF (Figure 3E) was difficult to observe. Monosodium phosphate ionizes to sodium cations (Na^+^) and dihydrogen phosphate anions (H_2_PO_4_^−^) when it dissolves in water. On the other hand, disodium phosphate ionizes to sodium cations and hydrogen phosphate anions (HPO_4_^2−^). The formation of V-CAp with PS-2 is more rapid than with other tested solutions with the same immersing time, as the dihydrogen phosphate anions were less favorable to form carbonated apatite compared to the hydrogen phosphate anions [43]. In addition, CCV can also transform into calcite, which is the stable state of calcium carbonate, as observed in Figure 3A,D [44].

**Figure 4:**
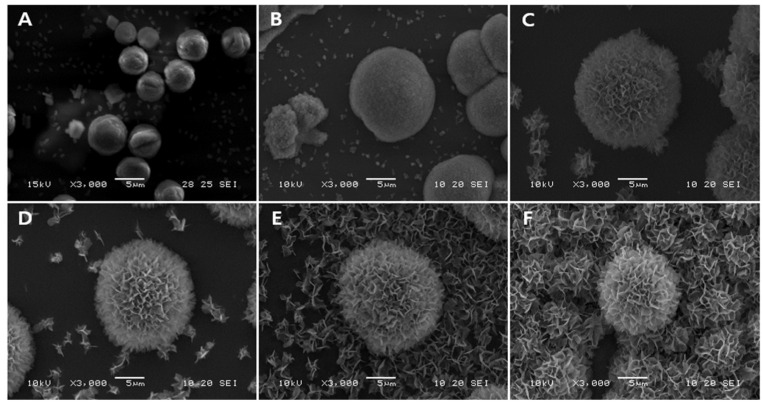
SEM images of V-CAp formation from CCV over time: (**A**) 0 h, (**B**) 2 h, (**C**) 4 h, (**D**) 8 h, (**E**) 12 h, and (**F**) 16 h. The glass was coated CCV first and immersed in PS-2 solution at 37 °C.

The time required for apatite carbonate generation on a template or scaffold is also crucial for bone grafting. In cases of bone defects, it is essential for the scaffold to become bioactive within a relatively short period of time for use in surgery. The transformation of V-CAp from CCV over time was observed through SEM images (Figure 4). After immersion for 2 h, the size of CCV slightly increased, and the surface roughness changed. As mentioned earlier, CCV sizes of 4–6 μm increased to 5–16 μm and up to 22 μm. These results demonstrated that CCV with PS-2 could form V-CAp within 16 h. The surface roughness was calculated by using Image J software. The average surface of CCV, which was 6.74 μm^2^, increased up to 19.72 μm^2^ after the V-CAp was immersed for 16 h. The observed increase in particle size and average surface roughness can be attributed to the deposition and growth of V-CAp. The SEM images showed that CCV became rough and irregular after immersion in PS-2. This change in morphology is due to the deposition of CAp crystals, which form agglomerated structures on the particle surface. As V-CAp crystals nucleate and grow, they coalesce into larger aggregates, resulting in improved surface roughness. In addition, the continuous deposition of V-CAp crystals results in an overall increase in particle size. This process is consistent with the Ostwald ripening phenomenon, where smaller grains dissolve and larger grains grow, resulting in an increase in average grain size and surface roughness [45,46].

### 3.2. Characterization of Generated V-CAp

**Figure 5:**
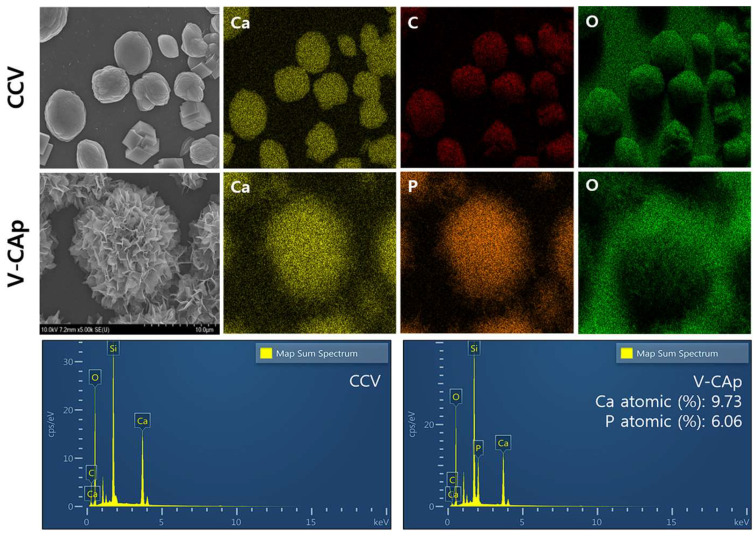
The composition of prepared CCV and V-CAp was analyzed using SEM-EDS. The CCV consisted of Ca, C, and O elements, which are the main components of calcium carbonate. The V-CAp consists of Ca, P, and O elements, which are the main components of carbonated apatite. The Ca and P ion’s atomic percentages were 9.73% and 6.06%, respectively (Ca/P mole ratio = 1.61).

The SEM-EDS was used to analyze the elemental composition and content of V-CAp (Figure 5). The EDS elemental maps for CCV showed the presence of calcium (Ca), carbon (C), and oxygen(O), with no detectable phosphorus (P), indicating that the original particles are composed of pure calcium carbonate. For V-CAp, the elemental maps revealed the presence of Ca, P, and O, the main components of carbonated apatite, confirming the incorporation of phosphate into the V-CAp. The EDS spectra for CCV demonstrated the peak for Ca, C, and O, consistent with the composition of calcium carbonate. The V-CAp spectra also showed the peak of Ca, C, and O and a prominent peak for phosphorus, indicating the formation of a calcium phosphate phase [47]. Pure hydroxyapatite, a main component of bone, has a Ca/P mole ratio of 1.67, and the Ca/P ratio of carbonated apatite ranges from 1.5 to 1.67 [48]. The V-CAp contains 9.73% atomic Ca and 6.06% atomic P, which supports the formation of carbonated apatite. The Ca/P mole ratio of V-CAp was 1.61, which is in the range of carbonated apatite. The transformation of calcium carbonate into carbonated apatite (V-CAp) through immersion in PS-2 is evidenced by elemental changes observed in the SEM-EDS analyses. It is possible to explain this process through the dissolution–reprecipitation process [49]. The immersion of CCV in PS-2 leads to the partial dissolution of calcium carbonate, releasing Ca^2+^ and CO_3_^2−^ ions into the solution, and these ions then interact with phosphate ions (PO_4_^3−^) in the solution, resulting in the nucleation and growth of V-CAp on the surface of the CCV. The SEM-EDS results demonstrate that CCV in PS-2 effectively transforms into the V-CAp, characterized by the incorporation of phosphate. This transformation enhances the material’s potential for biomedical applications, particularly in bone tissue engineering, due to its compositional similarity to natural bone and improved bioactivity.

**Figure 6:**
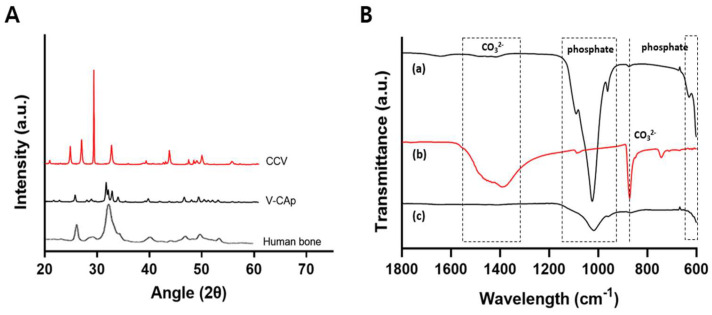
(**A**) CCV, V-CAp, and human bone were analyzed using XRD. (**B**) The functional groups of hydroxyapatite (a), CCV (b), and V-CAp (c) were analyzed through ATR-FTIR. The wavelength ranges of 550–600 and 960–1120 cm^−1^ were the represented phosphate group. The peak of 743 cm^−1^, 872 cm^−1^ and the wavelength range 1319–1470 cm^−1^ were the represented carbonate group.

Figure 6A shows the XRD patterns of CCV and V-CAp on the glass surface. In the 20°–60° range, the XRD patterns revealed the presence of vaterite at 24.9°, 27.1°, and 32.7° in 2θ, corresponding to crystal planes (100), (101), and (102), respectively. Also, a strong peak of hydroxyapatite was observed, with the representative reflecting crystal planes at 25.7°, 31.71°, 32.1°, 32.9°, 46.6°, and 49.3°. According to the PDF (Powder Diffraction File) database of the ICDD (International Centre for Diffraction Data), the peaks of V-CA p represent the carbonated apatite. The XRD analysis showed a decrease in the peak of calcium carbonate and the appearance of a carbonated apatite peak, demonstrating that the conversion of CCV to V-CAp was sufficient to immerse PS-2. At this point, structural changes in the biomineral and SEM-EDS (chemical changes already demonstrated) confirmed that all of the CCV had been converted to V-CAp. This suggests that calcium carbonate was transformed into carbonated apatite. In addition, the XRD pattern of V-CAp closely resembles that of human bone, suggesting that the synthetic process yields a product with similar crystallographic properties to natural bone apatite.

The functional groups of V-CAp were determined using ATR-FTIR (Figure 6B). Most of the bands were characteristic of phosphate groups (at 550–600 and 960–1120 cm^−1^) [50]. The presence of carbonate substituted in the apatite structure was indicated by strong carbonate peaks at 743 cm^−1^, 872 cm^−1^, and in the range of 1319–1470 cm^−1^. The peak at 872 cm^−1^ could be attributed to HPO_4_, which is characteristic of carbonated apatite [49]. The decrease in the carbonate peak at 872 cm^−1^ was attributed to the conversion of CCV to V-CAp. In addition, the bands within the phosphate region, ranging from 960 to 1120 cm^−1^, showed increased intensity after the formation of V-CAp. The transformation of calcium carbonate (CCV) into carbonated apatite (V-CAp) through immersion in phosphate solution is confirmed by both XRD and FT-IR analyses. These results provide detailed insights into the structural and compositional changes occurring during the transformation process.

Overall, our findings demonstrated that the V-CAp transformed from CCV exhibited elemental composition, chemical properties, and structural features similar to hydroxyapatite. V-CAp could provide a bone-like environment similar to hydroxyapatite through surface biocoating.

### 3.3. In Vitro Proliferation and ALP Activity on the V-CAp Coated Surface

**Figure 7:**
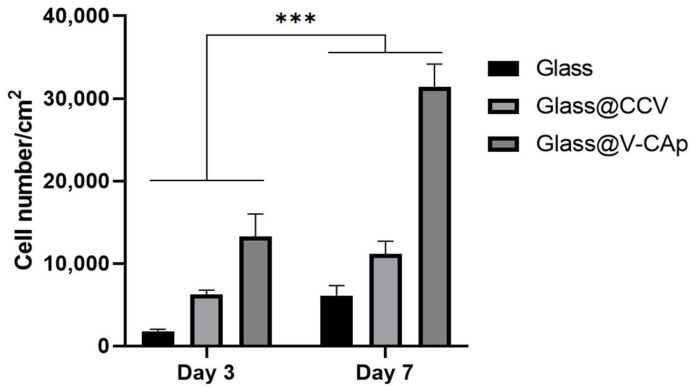
MC3T3-E1 cell proliferation after 3 and 7 days of culturing on glass with and without CCV and V-CAp. Cell proliferation was analyzed using the MTS assay. The cells were incubated at 37 °C in a 5% CO_2_ incubator for 3 and 7 days. The growth media was replaced every 3 days. Error bars represent the SD obtained from three replicates. *** *p* < 0.001.

The results of the cell proliferation experiments provide key insights into the biocompatibility and bioactivity of the materials under study. In particular, the proliferation of cells on the non-bioactivity of glass surfaces and the transformed carbonated apatite (V-CAp) surfaces was assessed in order to determine the impact of surface modification on cellular behavior. Figure 7 shows the proliferation of cells cultured for 3 and 7 days on glass with and without CCV and V-CAp. As previously reported [51,52], an implant’s surface properties and surface chemistry will influence cell response at the cell–material interface, ultimately affecting the rate and quality of new-tissue formation. This study demonstrated that cell proliferation on surface coatings was significant during the culture period. While the difference in cell proliferation on glass and CCV-coated surfaces was not significant, cells on V-CAp-coated glass exhibited a higher proliferation rate compared to the other groups on day 3. Furthermore, on day 7, cell proliferation was significantly higher on V-CAp-coated glass compared to day 3. V-CAp resulted in a 2.3-fold increase in proliferation compared to glass. This enhancement can be attributed to the increased surface roughness and the presence of both carbonate ions and phosphate ions on the V-CAp surface, as confirmed by EDS mapping and FT-IR analysis. The presence of phosphate ions, in particular, is known to enhance osteoblast activity and promote better cell attachment, spreading, and proliferation. The surface roughness further supports cell proliferation by providing a more favorable microenvironment for cell adhesion [53]. The structural and compositional similarity of V-CAp to natural bone minerals (as demonstrated by XRD and FT-IR analyses) suggests that the material’s bioactivity is closely aligned with the physiological needs of bone cells. This biomimetic property likely contributes to the observed increase in cell proliferation.

**Figure 8:**
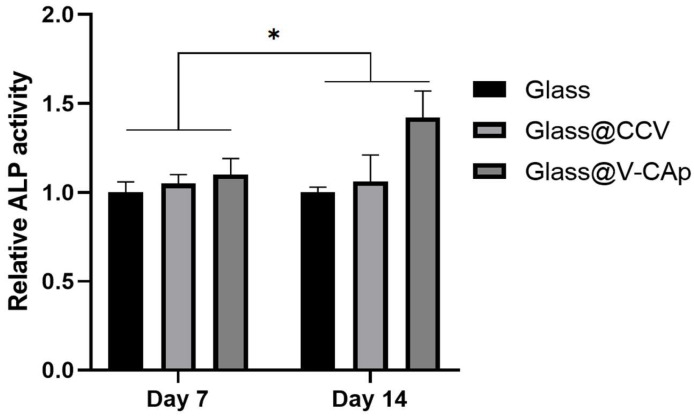
ALP activity of MC3T3-E1 cells on glass with and without CCV and V-CAp. The cell differentiation was analyzed using the ALP assay. The cells were incubated at 37 °C in a 5% CO_2_ incubator for 1 week. The medium was changed to a differentiation medium (same as above), and the ALP activity was measured after 7 and 14 days. The growth media and differentiation media were replaced every 3 days. Error bars represent the SD obtained from three replicates. * *p* < 0.05.

The cell differentiation studies provide insights into the bioactivity of the studied materials and their potential for bioapplications. ALP activity was measured to compare the effects of different surfaces on osteoblastic differentiation by culturing MC3T3-E1 cells for 7 and 14 days on pure glass, CCV-coated glass, and V-CAp-coated glass (Figure 8). Similar to the results of the MTS assay, ALP activity increased gradually over time. As shown in Figure 6, the ALP activities of the MC3T3-E1 cells cultured on pure glass, CCV, and V-CAp did not show a significant difference on day 7. However, the ALP activity in the V-Cap-coated group was 1.4-fold higher compared to the pure glass group on day 14. The study by Xun et al. demonstrated that CAp-formed scaffolds via SBF immersion promoted bone tissue formation [54]. Our findings are consistent with the osteogenic differentiation of V-CAp coated by conversion from CCVs, which is a conventional biomimetic method of CAp formation. Although these results do not definitively indicate that the regenerative functions of bone for V-CAp are identical to those of a bone implant, the V-CAp coating may support the induction of osteogenic differentiation rather than a non-bioactive surface.

## 4. Conclusions

The scaffolds or supports used today for bone tissue regeneration are not bioactive and require a long time to regenerate. To overcome this disadvantage, researchers have been working to induce bone regeneration by adding bioactivity. In this study, we proposed a step-wise biomimetic method to make carbonated apatite (CAp) transformed from calcium carbonate vaterite (CCV). There was a variety of methods to form and apply CAp to non-bioactive bone grafts. Among the methods, the incubation of the surface material in SBF solution was the most widely used method, but it requires a long reaction time (over 1 week). Immersion of CCV in PS-2 (disodium phosphate solution with pH 7.6 adjusted) facilitated the formation of CCV-transformed into CAp (V-CAp), which shares similarities with hydroxyapatite found in real bone. As a result, the SEM images showed the transformation of CCV into V-CAp, and the time-dependent formation of V-CAp could be achieved in 4–16 h. Furthermore, the surface coated with V-CAp showed bioactivity to mediate osteoblast cell proliferation and ALP activity. This study highlights that the step-wise formation of V-CAp is a more efficient biomimetic strategy to obtain bioactive surfaces, which are critical for bone graft or scaffold design. Furthermore, the bioactivity of scaffolds and supports has the potential to play an important role in bone therapy.

## Figures and Tables

**Table 1:** The phosphates in different types of phosphate solutions.

Phosphate Solution	Phosphate Ingredient
P buffer (pH 7.6)	Monosodium phosphate, disodium phosphate
PS-1 (Adjusted pH 7.6)	Monosodium phosphate
PS-2 (Adjusted pH 7.6)	Disodium phosphate
Commercial PBS (pH 7.6)	Monosodium phosphate, disodium phosphate
SBF (pH 6.7)	Disodium phosphate

P, phosphate; PS, phosphate solution; PBS, phosphate buffer saline; SBF, simulated body fluid.

## Data Availability

The dataset is available on request from the authors.

## References

[1] Taton T.A. (2001). Boning up on biology. Nature.

[2] Bigham-Sadegh A., Oryan A. (2014). Basic concepts regarding fracture healing and the current options and future directions in managing bone fractures. Int. Wound J..

[3] Hung K.-Y., Lo S.-C., Shih C.-S., Yang Y.-C., Feng H.-P., Lin Y.-C. (2013). Titanium surface modified by hydroxyapatite coating for dental implants. Surf. Coat. Technol..

[4] Feng P., Zhao R., Tang W., Yang F., Tian H., Peng S., Pan H., Shuai C. (2023). Structural and Functional Adaptive Artificial Bone: Materials, Fabrications, and Properties. Adv. Funct. Mater..

[5] Shuai C., Yang W., Feng P., Peng S., Pan H. (2020). Accelerated degradation of HAP/PLLA bone scaffold by PGA blending facilitates bioactivity and osteoconductivity. Bioact. Mater..

[6] Yang Y., Zhang Q., Xu T., Zhang H., Zhang M., Lu L., Hao Y., Fuh J., Zhao X. (2020). Photocrosslinkable nanocomposite ink for printing strong, biodegradable and bioactive bone graft. Biomaterials.

[7] Zhao Y., Chen J., Zou L., Xu G., Geng Y. (2019). Facile one-step bioinspired mineralization by chitosan functionalized with graphene oxide to activate bone endogenous regeneration. Chem. Eng. J..

[8] Guo F., Wang E., Yang Y., Mao Y., Liu C., Bu W., Li P., Zhao L., Jin Q., Liu B. (2023). A natural biomineral for enhancing the biomineralization and cell response of 3D printed polylactic acid bone scaffolds. Int. J. Biol. Macromol..

[9] Von Euw S., Wang Y., Laurent G., Drouet C., Babonneau F., Nassif N., Azaïs T. (2019). Bone mineral: New insights into its chemical composition. Sci. Rep..

[10] Ariani M.D., Salim S. (2015). In vitro and in vivo evaluation of carbonate apatite-collagen scaffolds with some cytokines for bone tissue engineering. J. Indian Prosthodont. Soc..

[11] Mondal S., Pal U., Dey A. (2016). Natural origin hydroxyapatite scaffold as potential bone tissue engineering substitute. Ceram. Int..

[12] Jayasree R., Madhumathi K., Rana D., Ramalingam M., Nankar R.P., Doble M., Kumar T.S.S. (2018). Development of Egg Shell Derived Carbonated Apatite Nanocarrier System for Drug Delivery. J. Nanosci. Nanotechnol..

[13] Kajander K., Sirkiä S.V., Vallittu P.K., Heino T.J., Määttä J.A. (2023). Bioactive glasses promote rapid pre-osteoblastic cell migration in contrast to hydroxyapatite, while carbonated apatite shows migration inhibiting properties. Sci. Rep..

[14] Mochizuki C., Hara H., Takano I., Hayakawa T., Sato M. (2013). Application of carbonated apatite coating on a Ti substrate by aqueous spray method. Mater. Sci. Eng. C.

[15] Kong L.B., Ma J., Boey F. (2002). Nanosized hydroxyapatite powders derived from coprecipitation process. J. Mater. Sci..

[16] Liu D.-M., Troczynski T., Tseng W.J. (2001). Water-based sol–gel synthesis of hydroxyapatite: Process development. Biomaterials.

[17] Padmanabhan S.K., Haq E.U., Licciulli A. (2014). Rapid synthesis and characterization of silicon substituted nano hydroxyapatite using microwave irradiation. Curr. Appl. Phys..

[18] Liang W., Zhan L., Piao L., Rüssel C. (2011). Lead and copper removal from aqueous solutions by porous glass derived calcium hydroxyapatite. Mater. Sci. Eng. B.

[19] Midorikawa K., Hiromoto S., Yamamoto T. (2024). Carbonate content control in carbonate apatite coatings of biodegradable magnesium. Ceram. Int..

[20] Earl J.S., Wood D.J., Milne S.J. (2006). Hydrothermal synthesis of hydroxyapatite. J. Phys. Conf. Ser..

[21] Minh D.P., Tran N.D., Nzihou A., Sharrock P. (2014). Novel one-step synthesis and characterization of bone-like carbonated apatite from calcium carbonate, calcium hydroxide and orthophosphoric acid as economical starting materials. Mater. Res. Bull..

[22] Goldberg M.A., Donskaya N.O., Valeev D.V., Fomin A.S., Murzakhanov F.F., Leonov A.V., Konovalov A.A., Antonova O.S., Shoppert A.A., Kudryavtsev E.A. (2024). Mesoporous molybdate-substituted hydroxyapatite nanopowders obtained via a hydrothermal route. Ceram. Int..

[23] Frank-Kamenetskaya O., Kol’tsov A., Kuz’mina M., Zorina M., Poritskaya L. (2011). Ion substitutions and non-stoichiometry of carbonated apatite-(CaOH) synthesised by precipitation and hydrothermal methods. J. Mol. Struct..

[24] Tonegawa T., Ikoma T., Suetsugu Y., Igawa N., Matsushita Y., Yoshioka T., Hanagata N., Tanaka J. (2010). Thermal expansion of type A carbonate apatite. Mater. Sci. Eng. B.

[25] Li M., Wu G., Wang M., Hunziker E.B., Liu Y. (2022). Crystalline Biomimetic Calcium Phosphate Coating on Mini-Pin Implants to Accelerate Osseointegration and Extend Drug Release Duration for an Orthodontic Application. Nanomaterials.

[26] Kokubo T. (1990). Surface chemistry of bioactive glass-ceramics. J. Non-Cryst. Solids.

[27] Duan H., Cao C., Wang X., Tao J., Li C., Xin H., Yang J., Song Y., Ai F. (2020). Magnesium-alloy rods reinforced bioglass bone cement composite scaffolds with cortical bone-matching mechanical properties and excellent osteoconductivity for load-bearing bone in vivo regeneration. Sci. Rep..

[28] Mythili P., Madalina P., Roxana P., Alain L., Francesco B. (2017). Fabrication Methodologies of Biomimetic and Bioactive Scaffolds for Tissue Engineering Applications. Materials, Technologies and Clinical Applications.

[29] Li M., Wang M., Wei L., Werner A., Liu Y. (2023). Biomimetic calcium phosphate coating on medical grade stainless steel improves surface properties and serves as a drug carrier for orthodontic applications. Dent. Mater..

[30] Feng B., Chen J., Qi S., He L., Zhao J., Zhang X. (2001). Carbonate apatite coating on titanium induced rapidly by precalcification. Biomaterials.

[31] Leena M., Rana D., Webster T.J., Ramalingam M. (2016). Accelerated synthesis of biomimetic nano hydroxyapatite using simulated body fluid. Mater. Chem. Phys..

[32] Chaka A.M. (2018). Ab Initio Thermodynamics of Hydrated Calcium Carbonates and Calcium Analogues of Magnesium Carbonates: Implications for Carbonate Crystallization Pathways. ACS Earth Space Chem..

[33] Ferreira A.M., Vikulina A.S., Volodkin D. (2020). CaCO_3_ crystals as versatile carriers for controlled delivery of antimicrobials. J. Control. Release.

[34] Min K.H., Kim D.H., Pack S.P. (2024). Size Control of Biomimetic Curved-Edge Vaterite with Chiral Toroid Morphology via Sonochemical Synthesis. Biomimetics.

[35] Morse J.W., Arvidson R.S., Lüttge A. (2007). Calcium Carbonate Formation and Dissolution. Chem. Rev..

[36] Donatan S., Yashchenok A., Khan N., Parakhonskiy B., Cocquyt M., Pinchasik B.-E., Khalenkow D., Möhwald H., Konrad M., Skirtach A. (2016). Loading Capacity versus Enzyme Activity in Anisotropic and Spherical Calcium Carbonate Microparticles. ACS Appl. Mater. Interfaces.

[37] Wang J., Chen J.-S., Zong J.-Y., Zhao D., Li F., Zhuo R.-X., Cheng S.-X. (2010). Calcium Carbonate/Carboxymethyl Chitosan Hybrid Microspheres and Nanospheres for Drug Delivery. J. Phys. Chem. C.

[38] Li X., Yang X., Liu X., He W., Huang Q., Li S., Feng Q. (2018). Calcium carbonate nanoparticles promote osteogenesis compared to adipogenesis in human bone-marrow mesenchymal stem cells. Prog. Nat. Sci..

[39] Vikulina A., Webster J., Voronin D., Ivanov E., Fakhrullin R., Vinokurov V., Volodkin D. (2020). Mesoporous additive-free vaterite CaCO_3_ crystals of untypical sizes: From submicron to Giant. Mater. Des..

[40] Minh D.P., Nzihou A., Sharrock P. (2014). Carbonated hydroxyapatite starting from calcite and different orthophosphates under moderate hydrothermal conditions: Synthesis and surface reactivity in simulated body fluid. Mater. Res. Bull..

[41] Jalota S., Bhaduri S.B., Tas A.C. (2006). Effect of carbonate content and buffer type on calcium phosphate formation in SBF solutions. J. Mater. Sci. Mater. Med..

[42] Wong S.L., Deymier A.C. (2023). Phosphate and buffer capacity effects on biomimetic carbonate apatite. Ceram. Int..

[43] Minh D.P., Lyczko N., Sebei H., Nzihou A., Sharrock P. (2012). Synthesis of calcium hydroxyapatite from calcium carbonate and different orthophosphate sources: A comparative study. Mater. Sci. Eng. B.

[44] Sandin K., Kloo L., Nevsten P., Wallenberg R.L., Olsson L.-F. (2009). Formation of carbonated apatite particles from a supersaturated inorganic blood serum model. J. Mater. Sci. Mater. Med..

[45] Ibsen C.J.S., Chernyshov D., Birkedal H. (2016). Apatite Formation from Amorphous Calcium Phosphate and Mixed Amorphous Calcium Phosphate/Amorphous Calcium Carbonate. Chem. A Eur. J..

[46] Myszka B., Schüßler M., Hurle K., Demmert B., Detsch R., Boccaccini A.R., Wolf S.E. (2019). Phase-specific bioactivity and altered Ostwald ripening pathways of calcium carbonate polymorphs in simulated body fluid. RSC Adv..

[47] Jaramillo-Martínez S., Vargas-Requena C., Rodríguez-Gónzalez C., Hernández-Santoyo A., Olivas-Armendáriz I. (2019). Effect of extrapallial protein of Mytilus californianus on the process of in vitro biomineralization of chitosan scaffolds. Heliyon.

[48] Pastero L., Bruno M., Aquilano D. (2017). About the Genetic Mechanisms of Apatites: A Survey on the Methodological Approaches. Minerals.

[49] Benataya K., Lakrat M., Elansari L., Mejdoubi E. (2020). Synthesis of B-type carbonated hydroxyapatite by a new dissolution-precipitation method. Mater. Today Proc..

[50] Zhong Q., Li W., Su X., Li G., Zhou Y., Kundu S.C., Yao J., Cai Y. (2016). Degradation pattern of porous CaCO_3_ and hydroxyapatite microspheres in vitro and in vivo for potential application in bone tissue engineering. Colloids Surf. B Biointerfaces.

[51] Al-Hamdan S.H., Al-Hamdan K., Junker R., A Jansen J. (2012). Effect of implant surface properties on peri-implant bone healing: Implant stability and microcomputed tomographic analysis. Int. J. Oral Maxillofac. Implant..

[52] Kubies D., Himmlová L., Riedel T., Chánová E., Balík K., Douděrová M., Bártová J., Pešáková V. (2011). The interaction of osteoblasts with bone-implant materials: 1. The effect of physicochemical surface properties of implant materials. Physiol. Res..

[53] Li L., Crosby K., Sawicki M., Shaw L.L., Wang Y. (2012). Effects of Surface Roughness of Hydroxyapatite on Cell Attachment and Proliferation. J. Biotechnol. Biomater..

[54] Xun X., Li Y., Ni M., Xu Y., Li J., Zhang D., Chen G., Ao H., Luo H., Wan Y. (2024). Calcium crosslinked macroporous bacterial cellulose scaffolds with enhanced in situ mineralization and osteoinductivity for cranial bone regeneration. Compos. Part B Eng..

